# Tea consumption may improve psychological resilience among older adults with chronic diseases: a prospective cohort study

**DOI:** 10.3389/fpsyt.2025.1594067

**Published:** 2025-06-06

**Authors:** Huihe Chen, Ming Gao, Lanhui Huang, Xuehai Guan, Yuanfei Wei

**Affiliations:** ^1^ Department of Emergency, Wuming Hospital of Guangxi Medical University, Naning, China; ^2^ Department of Orthopaedics, The First People’s Hospital of Yunnan Province, Kunming, China; ^3^ Department of Geriatric Endocrinology and Metabolism, The First Affiliated Hospital of Guangxi Medical University, Naning, China; ^4^ Department of Anaesthesiology, the First Affiliated Hospital of Guangxi Medical University, Naning, China

**Keywords:** aged, health education, healthy lifestyle, multimorbidity, resilience, psychological

## Abstract

**Objective:**

To investigate the association between tea consumption and the dynamic change in psychological resilience (PR) among older adults with chronic diseases.

**Study design:**

A prospective cohort study.

**Methods:**

A total of 26,454 adults aged 60 and older from the Chinese Longitudinal Healthy Longevity Study were analysed. Tea consumption and PR were evaluated through survey at baseline and at the 3-year follow-up using drinking frequency and a validated scoring scale. Clustering analysis was used to identify multimorbidity clusters. Multivariable analysis was employed to investigate the association between tea consumption and PR change. Several sensitivity analyses were conducted.

**Results:**

The sample had an average age of 85.6 ± 12.0 years, with 55.7% female. Ten distinct multimorbidity clusters were identified. Daily tea drinkers exhibited greater improvement in PR (OR=1.176, 95% CI: 1.043-1.327) compared to non-drinkers over a 3-year follow-up. Females (OR=1.362, 95% CI: 1.124-1.649) and participants in the younger-old group (age < 85 years, OR=1.243, 95% CI: 1.075-1.436) were more likely to experience this benefit. This positive association remained significant in participants with multimorbidity (OR=1.437, 95% CI: 1.116-1.850), but not in those considered robust (OR=1.102, 95% CI: 0.931-1.304) or with a single chronic disease (OR=1.117, 95% CI: 0.878-1.421). Specifically, this association was most pronounced within the multimorbidity cluster characterized by cardiometabolic conditions (OR=3.902, 95% CI: 1.081-14.084). The results remained consistent across numerous sensitivity analyses.

**Conclusions:**

Daily tea consumption is positively associated with PR improvement among older adults, particularly those with cardiometabolic multimorbidity. Promoting tea drinking habit may represent a viable strategy for promoting active health during late life stages.

## Introduction

Chronic diseases (CDs) represent a significant source of stress for older adults (1, 2). Approximately one-fifth of the global disease burden arises in individuals aged 60 years and older, with CDs contributing significantly (3). Alongside CDs, chronic stress inevitably develops and can lead to episodes of depression, anxiety, or other psychological distress (4, 5). These adverse effects are exacerbated in older adults with multimorbidity (6–8), defined as having two or more coexisting CDs. As human lifespan increases, chronic stress following CDs not only affects individual quality of life in late years but also imposes a significant burden on the healthcare system (3, 9, 10). Therefore, finding cost-effective strategies to manage stress in older adults with CDs is crucial for promoting active health.

The Lifecourse Model of Multimorbidity Resilience (LMMR) captures the dynamic interplay of multidimensional factors that modulate adaptation to multimorbidity and disablement processes in later life (11). Within this framework, psychological resilience (PR) emerges as a central determinant of both functional preservation and recovery processes in chronic illness management (12). Evidence indicates that strong PR can alleviate psychological distress and therefore aid patients in coping with CDs (13–18). Intriguingly, healthy lifestyles, including fruit/vegetable consumption and regular physical activities, are found to be associated with a high level of PR (19). In older adults with multimorbidity, other lifestyles such as smoking, sleep patterns, and appetite are also significantly associated with PR across various multimorbidity clusters (11, 20). For example, non-smoking is linked to a higher level of resilience in the cardiovascular/metabolic multimorbidity cluster, whereas a sedentary lifestyle is associated with a lower level of resilience in the mental-health cluster. However, this evidence cannot establish a causal relationship due to the cross-sectional study design. Moreover, prior research on PR has been limited to single-time-point assessments and does not account for the dynamic changes in PR over time. Therefore, additional longitudinal studies on lifestyle behaviors are needed to explore potential modifiable factors contributing to the dynamic change in PR.

Tea drinking is a widespread global habit. Reportedly, tea is the most consumed beverage after water, due to its aroma, affordability and ease of preparation (21). In addition to these qualities, tea has health benefits. Research has shown that tea consumption may lower the risk of mental illness, especially depression and anxiety, and improve quality of life (22–24). As another aspect of mental health different from depression and anxiety, it is worth asking whether tea drinking habit is also associated with PR. However, a cross-sectional study among older adults found no association between tea consumption and PR (25). This contrasts with the earlier health-related findings regarding tea consumption and does not consider the coexistence of CD/multimorbidity. Thus, the relationship between tea consumption and the dynamic change in PR needs to be further explored and clarified, especially in different CD/multimorbidity categories.

Using the Chinese Longitudinal Healthy Longevity Survey (CLHLS), we aim to (1) investigate the longitudinal association between tea consumption and the dynamic changes in PR (2), ascertain their relationship across various disease contexts. We hypothesized that regular tea consumption would improve PR, particularly in the presence of multimorbidity.

## Methods

### Study population

The longitudinal data from CLHLS was utilized in this study. The CLHLS recruits representative community older adults from 23 out of the 31 provinces in China to investigate healthy longevity (26). It has seven waves of follow-up surveys since 1998. The 1998 wave was excluded because of missing information on PR. Data from all participants in the 2002 wave and the newly recruited participants in the 2005, 2008, 2011, 2014, and 2018 waves were included. Eligible participants were those aged 60 years and older, with evaluation of PR, tea consumption, and records of ten chronic diseases. At baseline, 26,454 participants were enrolled. During the follow-up period, 12,650 participants were lost or had died, and an additional 1,805 participants were further excluded due to missing values of PR and tea consumption. Finally, the 3-year follow-up sample consisted of 12,065 participants. The sampling process and study design are illustrated in **Supplementary Figure S1**. This study was approved by the Peking University Ethical Committee (IRB00001052-13074).

### Psychological resilience assessment

According to previous studies (16, 27), we assessed PR using a scale of five items. The questions (Qs) are as followings: (Q1) “Do you feel the older you get, the more useless you are?” (Q2) “Do you often feel fearful or anxious?” (Q3) “Do you often feel lonely and isolated?” (Q4) “Do you always look on the bright side of things?” (Q5) “Can you make your own decisions concerning your personal affairs?” The response to each item were scored on a five-point scale (always, often, sometimes, seldom, and never). The total PR score (PRS) ranged from 5-25. A higher score represents better PR. The detailed scoring method is presented in **Supplementary Table S1**. The PRS was measured at baseline and follow-up, respectively. Participants were categorized by two ways (1): better PR group (PRS ≥ median) vs. worse PR group (PRS < median, as reference) in cross-sectional analysis (2); PR improved group (changes in PRS>0) vs. PR declined group (changes in PRS ≤ 0, as reference) in longitudinal analysis.

### Tea consumption

Participants were categorized into four groups based on tea drinking habits: non-drinking, inconsistent drinking, consistent drinking, and daily drinking. To precisely assess habitual tea drinking, the following questions were asked: (Q1) “How often did you drink tea at around age 60?” and (Q2) “How often do you drink tea at present?” Both questions had the same response options in each wave. In consistence with responses in 2002-2005 waves, the response options in the 2008-2018 waves were collapsed into three categories: daily (consuming tea almost every day), occasionally (consuming tea not every day, but at least once per week or month), and rarely or never. Cross-sectionally, tea consumption habits were assessed based on Q1 and Q2. Participants who rarely or never drank tea for both target Qs were categorized into the “non-drinking” group. Similarly, participants who drank tea almost every day for both questions were categorized into the “daily drinking” group. Participants who rarely or never drank tea for either Q1 or Q2, but not in both Qs, were categorized into the “inconsistent drinking” group. The rest were categorized into the “consistent drinking” group. Longitudinally, participants were grouped using the same criteria as those used in the cross-sectional analysis, but only Q2 responses at baseline and the 3-year follow-up were considered as the target Qs.

### Covariates

Several variables were adjusted as covariates in regression analysis. Sociodemographic and socioeconomic characteristics included sex, age (the younger-old/the older-old, divided by 85 years), residential area (urban/rural), marital status (living with spouse/others), living arrangements (with household member/alone or in institution), education (illiteracy/literacy), occupation (agriculture/non-agriculture), pension (yes/no), and financial condition (sufficient/insufficient). Participants were divided into younger-old (aged below 85 years) and older-old (aged 85 years and older). Lifestyle included current habits of smoking (yes/no), alcohol consumption (yes/no), and exercising (yes/no). Physical function was assessed using the six items of basic activities of daily living (BADL) and the eight items of instrumental activities of daily living (IADL). The score of BADL and IADL ranged from 6-18 and 8-24, respectively, with a higher score representing a worse functional status. Cognitive function was assessed using the Chinese Version of the Mini-Mental State Examination (MMSE) scale, which contains 24 items with a total score of 30. A higher score of MMSE indicated a better cognitive function. The detail scoring methods were described in our previous work (28).

The medical history of ten most prevalent chronic conditions was collected through self-report questions, including hypertension, heart disease, diabetes mellitus (DM), cerebrovascular disease (CVD), respiratory disease, cancer, peptic ulcer, Parkinson’s disease, arthritis, and dementia. Each condition had a “yes/no” response. Participants were categorized into three groups based on the total number of concurrent conditions: zero chronic disease group, single chronic disease group, and multimorbidity group (with 2 or more chronic diseases).

### Statistical analysis

The count (percentage) and mean (standard deviation [SD]) of the covariates were displayed. Chi-square and t-tests were used to evaluate group differences. Three sets of logistic regression models were constructed to explore the cross-sectional and longitudinal relationships between tea consumption and better PR or PR improvement, respectively. Model 1 adjusted for age, sex, education, marital status, residential area, living arrangement, occupation, pension, financial condition, and lifestyle habits such as smoking, alcohol consumption, and exercising. Model 2 additionally adjusted for functional status, including BADL, IADL and MMSE scores. Model 3 was further adjusted for chronic conditions. To understand how habitual tea drinking impacts PR in different disease backgrounds, we conducted clustering analysis by using Partitioning around Medoids algorithm (29) to identify distinct multimorbidity cluster groups. Subsequently, regression analysis stratified by age, sex, and various disease contexts was performed. Sensitivity analysis involved (1): comparing the general characteristics of the follow-up participants with those who were died or lost to follow up (2); the changes of PRS at follow-up were used as dependent variable to repeat the main analyses (3); to avoid the impact of cognitive dysfunction, several subsets of the total sample were extracted by excluding participants with severe cognitive impairment (MMSE<21 and self-reported dementia) to testify our main findings. All statistical analyses were performed using SPSS (Version 23.0) and RStudio (Version 2022.02.3). A two-sided *p* < 0.05 was considered statistically significant.

## Results

### Characteristics of studied sample

**Figure 1:**
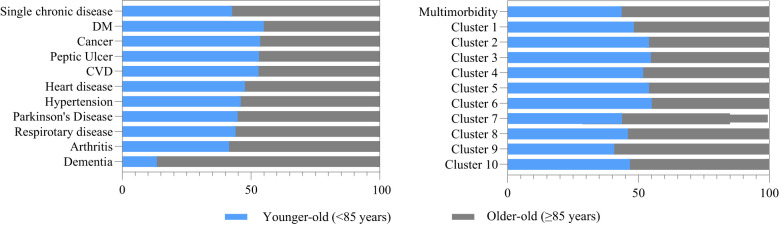
Disease distribution in different age group. DM: diabetes mellitus; CVD: cerebrovascular disease; Cluster 1: hypertension and respiratory disease; Cluster 2: hypertension and peptic ulcer; Cluster 3: hypertension and CVD; Cluster 4: hypertension and heart disease; Cluster 5: hypertension, heart disease, and DM; Cluster 6: hypertension, heart disease, and arthritis; Cluster 7: hypertension, respiratory disease, and arthritis; Cluster 8: hypertension, heart disease, respiratory disease, arthritis, and peptic ulcer; Cluster 9: hypertension, heart disease, DM, CVD, and respiratory disease; Cluster 10: hypertension, DM, heart disease, CVD, respiratory disease, cancer, peptic ulcer, Parkinson's disease, arthritis, dementia; DM, diabetes mellitus; CVD, cerebrovascular disease.

The overall sample had a mean age of 85.6 ± 12.0 years. The majority were female (55.7%). The mean PRS at baseline was 18.7 ± 3.2. Both medians of the PRSs were 19. Participants who drank tea daily were younger and had better PRS compared to those with other tea drinking habits (**Table 1**). Regarding chronic conditions, the older-old participants (≥85 years) consisted of larger proportions in single chronic disease groups, whereas the younger-old (<85 years) participants dominated in multimorbidity groups. Specifically, the prevalence of dementia among the older-old was 6.6 times of that in the younger-old (**Figure 1**). The characteristics of ten identified multimorbidity clusters are summarized in **Table 2**, with different main conditions in each group. For example, the most prevalent multimorbidity cluster included 10 conditions. Many other clusters were characterized by cardiometabolic conditions, such as Cluster 3, 4, and 5.

**Table 1:** Demographics by different tea drinking habits at baseline.

Variables	Overall n=26,454	Non-drinking n=11,597	Inconsistent drinking n= 4,416	Consistent drinking n=4,485	Daily drinking n=5,956
Age (mean ± SD)*	85.6 ± 12.0	86.5 ± 12.2	88.8 ± 10.4	84.7 ± 11.8	82.4 ± 12.2
Sex, female (n (%))*	14729 (55.7)	7666 (66.1)	2442 (55.3)	2257 (50.3)	2364 (39.7)
Education, illiteracy (n (%))*	15945 (60.5)	8023 (69.4)	2720 (61.9)	2496 (55.8)	2706 (45.5)
Marital status (n (%))*					
Living with spouse	13036 (49.3)	5581 (48.2)	1619 (36.7)	2282 (50.9)	3554 (59.7)
Others	13396 (50.7)	6008 (51.8)	2791 (63.3)	2199 (49.1)	2398 (40.3)
Residential area (n (%))*					
Urban area	11541 (43.6)	4472 (38.6)	2048 (46.4)	1994 (44.5)	3027 (50.8)
Rural area	14913 (56.4)	7125 (61.4)	2368 (53.6)	2491 (55.5)	2929 (49.2)
Living arrangement (n (%))*					
With household member	21702 (82.1)	9486 (81.8)	3514 (79.7)	3680 (82.1)	5022 (84.3)
Alone/In institution	4734 (17.9)	2104 (18.2)	894 (20.3)	802 (17.9)	934 (15.7)
Occupation (n (%)) *					
Agriculture	16320 (61.9)	7952 (68.8)	2689 (61.0)	2638 (59.0)	3041 (51.1)
Non-agriculture	10055 (38.1)	3598 (31.2)	1718 (39.0)	1831 (41.0)	2908 (48.9)
Pension, yes (n (%))*	5055 (19.1)	1515 (13.1)	747 (16.9)	942 (21.0)	1851 (31.1)
Financial condition (n (%))*					
Sufficient	21356 (80.8)	8977 (77.5)	3588 (81.3)	3774 (84.2)	5017 (84.2)
Insufficient	5074 (19.2)	2606 (22.5)	823 (18.7)	706 (15.8)	939 (15.8)
Smoking, yes (%) *	5104 (19.3)	1832 (15.8)	819 (18.6)	892 (19.9)	1561 (26.2)
Alcohol consumption, yes (%)*	5285 (20.0)	1924 (16.6)	912 (20.7)	899 (20.1)	1550 (26.1)
Exercising, yes (%)*	8057 (30.5)	3157 (27.3)	1357 (30.8)	1376 (30.7)	2167 (36.4)
BADL score (mean ± SD)*	6.8 ± 1.9	6.8 ± 1.9	7.1 ± 2.3	6.7 ± 1.9	6.5 ± 1.6
IALD score (mean ± SD)*	13.3 ± 5.7	13.8 ± 5.9	14.5 ± 5.9	12.8 ± 5.5	11.9 ± 5.2
MMSE score (mean ± SD)*	24.8 ± 5.9	24.3 ± 6.2	24.0 ± 6.1	25.1 ± 5.6	26.2 ± 5.0
Zero chronic disease (n (%))*	14068 (53.2)	6439 (55.5)	2336 (52.9)	2291 (51.1)	3002 (50.4)
Single chronic disease (n (%))*	6472 (24.5)	2857 (24.6)	1022 (23.1)	1092 (24.3)	1501 (25.2)
Multimorbidity (n (%))*	5914 (22.4)	2301 (19.8)	1058 (24.0)	1102 (24.6)	1453 (24.4)
PRS at baseline (mean ± SD)*	18.7 ± 3.2	18.5 ± 3.1	18.4 ± 3.1	18.6 ± 3.1	19.5 ± 3.2

BADL, basic activities of daily living; IADL, instrumental activities of daily living; MMSE, mini-mental state examination; PRS, psychological resilience score.

An asterisk (*) indicates statistically significant intergroup differences (p < 0.05).

**Table 2:** Characteristics of identified multimorbidity clusters.

Cluster	n	Hypertension (%)	DM (%)	Heart disease (%)	CVD (%)	Respiratory Disease (%)	Cancer (%)	Peptic ulcer (%)	Parkinson’s Disease (%)	Arthritis (%)	Dementia (%)	Main conditions	
1	804	541 (100)	61 (7.6)	207 (25.7)	89 (11.1)	776 (96.5)	34 (4.2)	128 (15.9)	20 (2.5)	123 (15.3)	34 (4.2)	Hypertension and respiratory disease
2	305	232 (76.1)	18 (5.9)	89 (29.2)	42 (13.8)	0 (0)	13 (4.3)	305 (100)	14 (4.6)	47 (15.4)	10 (3.3)	Hypertension and peptic ulcer
3	376	301 (80.1)	33 (8.8)	135 (35.9)	376 (100)	0 (0)	5 (1.3)	0 (0)	7 (1.9)	0 (0)	9 (2.4)	Hypertension and CVD
4	442	435 (98.4)	0 (0)	442 (100)	0 (0)	0 (0)	8 (1.8)	0 (0)	2 (0.5)	0 (0)	8 (1.8)	Hypertension and heart disease
5	392	354 (90.3)	392 (100)	247 (63)	0 (0)	0 (0)	20 (5.1)	21 (5.4)	6 (1.5)	0 (0)	9 (2.3)	Hypertension, heart disease, and DM
6	372	249 (66.9)	59 (15.9)	372 (100)	47 (12.6)	53 (14.2)	13 (3.5)	61 (16.4)	4 (1.1)	372 (100)	10 (2.7)	Hypertension, heart disease, and arthritis
7	554	554 (100)	44 (7.9)	52 (9.4)	83 (15)	333 (60.1)	18 (3.2)	145 (26.2)	145 (26.2)	554 (100)	33 (6.0)	Hypertension, respiratory disease, and arthritis
8	485	485 (100)	43 (8.9)	485 (100)	41 (8.5)	485 (100)	6 (1.2)	485 (100)	2 (0.4)	485 (100)	6 (1.2)	Hypertension, heart disease, respiratory disease, arthritis, and peptic ulcer
9	621	582 (93.7)	547 (88.1)	606 (97.6)	501 (80.7)	489 (78.7)	87 (14)	119 (19.2)	12 (1.9)	193 (31.1)	27 (4.3)	Hypertension, heart disease, DM, CVD, and respiratory disease
10	1563	1541 (98.6)	1529 (97.8)	1542 (98.7)	1538 (98.4)	1545 (98.8)	1529 (97.8)	1536 (98.3)	1498 (95.8)	1520 (97.2)	1494 (95.6)	Ten included chronic diseases

CVD, cerebrovascular disease; DM, diabetes mellitus.

### Relationships between tea drinking habits and psychological resilience

In cross-sectional analyses, daily tea consumption was associated with better PR. Compared with non-drinkers, the fully adjusted model showed that only daily tea drinkers had a higher chance of better PR (odd ratio [OR]=1.174, 95%CI: 1.089-1.266). The OR (95%CI) of inconsistent and consistent tea drinkers were 0.888 (0.818-0.963) and 0.824 (0.761-0.893), respectively. In longitudinal analyses, consistent and daily tea consumption significantly associated with PR improvement. Compared with non-drinkers, the ORs (95%CI) of consistent drinkers and daily drinkers in Model 1, Model 2, and Model 3 were 1.171 (1.046-1.311) vs 1.132 (1.008-1.272), 1.187 (1.056-1.334) vs 1.179 (1.046-1.33), and 1.183 (1.053-1.330) vs 1.176 (1.043-1.327), respectively. Discrepancies were found in different sex and age subgroups. In comparison with male participants, the longitudinal effect of tea consumption on PR was only statistically significant among female participants, with ORs (95%CI) of 1.19 (1.008, 1.406) in consistent drinking and 1.362 (1.124-1.649) in daily drinking. The younger-old (<85 years) were more likely to benefit from tea drinking habits for PR improvement: 1.154 (1.024-1.299) in inconsistent drinking, 1.303 (1.128-1.505) in consistent drinking, and 1.243 (1.075-1.436) in daily drinking. No statistically significant associations were found among the older-old participants (**Table 3**).

**Table 3:** Logistic regression models for tea consumption and PR.

Models	Inconsistent drinking OR (95%CI), *p*	Consistent drinking OR (95%CI), *p*	Daily drinking OR (95%CI), *p*
Better PR at baseline			
Model 1	0.874 (0.811, 0.941), <0.001	0.839 (0.78, 0.903), <0.001	1.223 (1.141, 1.312), <0.001
Model 2	0.881 (0.812, 0.956), <0.001	0.819 (0.756, 0.886), <0.001	1.169 (1.085, 1.260), <0.001
Model 3	0.888 (0.818, 0.963), 0.004	0.824 (0.761, 0.893), <0.001	1.174 (1.089, 1.266), <0.001
Female	0.917 (0.824, 1.02), 0.109	0.787 (0.707, 0.876), <0.001	1.061 (0.955, 1.178), 0.271
Male	0.869 (0.765, 0.987), 0.03	0.88 (0.78, 0.993), 0.038	1.292 (1.157, 1.443), <0.001
Younger-old (< 85 years)	0.85 (0.741, 0.974), 0.02	0.832 (0.74, 0.935), 0.002	1.297 (1.164, 1.446), <0.001
Older-old (≥ 85 year)	0.907 (0.819, 1.004), 0.061	0.83 (0.744, 0.926), 0.001	1.06 (0.954, 1.178), 0.275
PR improvement at 3-year follow-up			
Model 1	1.067 (0.976, 1.167), 0.156	1.171 (1.046, 1.311), 0.006	1.132 (1.008, 1.272), 0.037
Model 2	1.073 (0.978, 1.178), 0.137	1.187 (1.056, 1.334), 0.004	1.179 (1.046, 1.330), 0.007
Model 3	1.071 (0.976, 1.176), 0.149	1.183 (1.053, 1.330), 0.005	1.176 (1.043, 1.327), 0.008
Female	1.049 (0.929, 1.185), 0.440	1.19 (1.008, 1.406), 0.04	1.362 (1.124, 1.649), 0.002
Male	1.089 (0.941, 1.262), 0.253	1.176 (0.994, 1.391), 0.059	1.095 (0.93, 1.289), 0.274
Younger-old (< 85 years)	1.154 (1.024, 1.299), 0.018	1.303 (1.128, 1.505), <0.001	1.243 (1.075, 1.436), 0.003
Older-old (≥ 85 year)	0.953 (0.819, 1.109), 0.533	0.998 (0.816, 1.22), 0.982	1.088 (0.872, 1.357), 0.457

Model 1: adjusted for age, sex, illiteracy, marital status, residential area, living arrangement, occupation, pension, financial condition, smoking, drinking alcohol, exercising. Model 2: further adjusted BADL, IADL, and MMSE. Model 3: further adjusted chronic disease. BADL, basic activities of daily living; IADL, instrumental activities of daily living; MMSE, mini-mental state examination; PR, psychological resilience; OR, odds ratio; CI, confidence interval.

### Variations in different disease context

**Figure 2:**
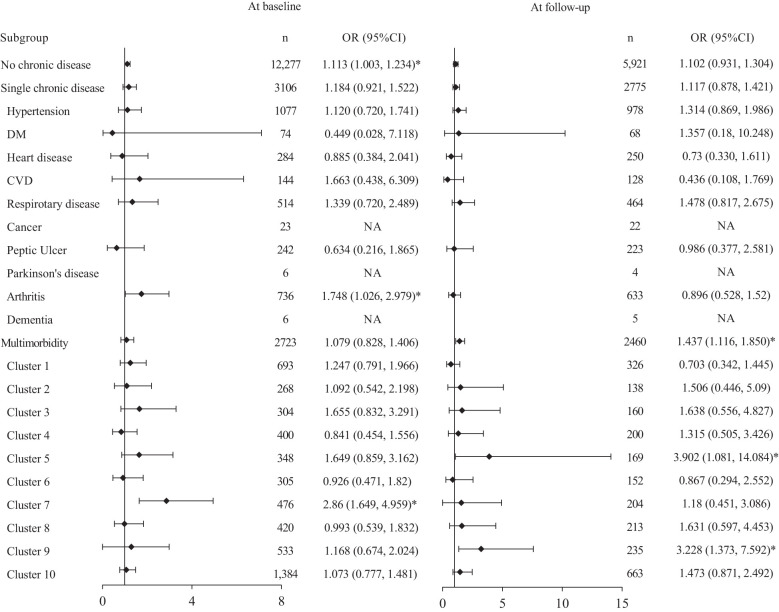
Forest plots of subgroup analysis in various disease contexts. Each model was adjusted for age, sex, illiteracy, marital status, residential area, living arrangement, occupation, pension, financial condition, smoking, drinking alcohol, exercising, BADL. IADL, and M.'Vl.SE. Dl\-1: diabetes mellitus: CVD: cerebrovascular disease; BADL: basic activities of daily living; IADL: instrumental activities of daily living; MMSE: mini-mental state examination; Cluster 1: hypertension and respiratory disease: Cluster 2: hypertension and peptic ulcer; Cluster 3: hypertension and C\ID; Cluster 4: hypertension and heart disease; Cluster 5: hypertension, heart disease, and DM; Clust.er 6: hypertension, heart disease, and arthritis; Cluster 7: hypertension, respiratory disease, and arthritis; Cluster 8: hj1Jertension, heart disease, respiratory disease, mthritis, and peptic ulcer; Cluster 9: hypertension, hemt disease, DM, CVD, atid respiratory disease; Cluster 10: hypertension, DM. heart disease, CVD, respiratory disease, cancer, peptic ulcer, Parkinson's disease, arthritis, and dementia; NA, not available. *:p <0.05.

Several sets of subgroup analyses were conducted to explore the effect of tea consumption in PR across different disease contexts (**Figure 2**). In participants without chronic disease, daily tea drinking was found significantly associated with PR cross-sectionally (OR=1.113, 95%CI: 1.003-1.234) but not longitudinally. In participants with single chronic disease, only daily tea drinkers with arthritis were more likely to have better PR (OR=1.748, 95%CI: 1.026-2.979). No statistically significant associations were found among those with only hypertension, DM, heart disease, CVD, respiratory disease, or peptic ulcer. The association among participants with only cancer, Parkinson’s disease, or dementia were not available because of scarce cases. In participants with multimorbidity, statistically significant association was found between daily tea drinking and PR improvement (OR=1.437, 95%CI: 1.116-1.850). Daily tea drinking was positively associated with PR in Cluster 7 characterized by hypertension, respiratory disease, and arthritis at baseline only (OR= 2.86, 95%CI: 1.649-4.959), but not at 3-year follow-up (OR=1.18, 95%CI: 0.451-3.086). Longitudinal subgroup analysis showed that Cluster 5, characterized by cardiometabolic conditions (hypertension, heart disease, and DM), gained the highest OR (3.902, 95%CI: 1.081-14.084) for PR improvement. Then followed by Cluster 9 characterized by additional coexistence of CVD and respiratory disease (OR=3.228, 95%CI: 1.373-7.592).

### Sensitivity analyses

In sensitivity analyses, participants lost to follow up shared similar characteristics with the total included sample except for older mean age and worse functional scores (Supplementary Materials, **Supplementary Table S2**). Regarding the relationships between daily tea drinking and PR, similar results were observed when using the changes of PRS as dependent variable (Supplementary Materials, **Supplementary Table S3**), as well as in a different studied sample excluding participants with severe cognitive impairment (Supplementary Materials, **Supplementary Table S4**).

## Discussion

This nationwide prospective cohort study uncovered a positive association between tea consumption and the dynamic change in PR among older adults over a 3-year follow-up period, especially among females and the younger-olds. This association was firstly evaluated across different disease contexts, revealing significant PR improvement with daily tea consumption only in participants with multimorbidity compared to individuals considered robust or with only one chronic disease. Specifically, this beneficial effect was most prominent in participants with cardiometabolic multimorbidity. The results were consistent across sensitivity analyses.

Habitual tea drinking, especially on a daily basis, may improve PR improvement over time in older adults. This large cohort of community-dwelling adults aged 60 years and older revealed a significant contribution of daily tea consumption to the improvement of PR over a 3-year follow-up period. These findings align with previous research indicating that lifestyle behaviors can enhance resilience (11, 20). Currently, research specifically focusing on the relationship between tea consumption and PR is scarce; only one published cross-sectional study reports conflicting results compared to ours, suggesting no statistically significant association between them (25). Nevertheless, studies on other aspects of mental health can serve as a reference. For instance, numerous prospective cohort studies have demonstrated that tea consumption can lower the risk of depression and enhance moods (30, 31). Additionally, variation in this association with age have been documented. One cross-sectional study observes a decreased risk of depressive symptoms in men after consuming green tea and in women after consuming floral tea (23). Regarding PR improvement, our study found a more pronounced positive impact of tea consumption in women compared to men. Although these results focus on different aspects of mental health, they collectively indicate that both women and men can achieve a favourable psychological outcome through tea consumption.

Notably, the PR benefits of daily tea consumption were statistically significant only among older adults with multimorbidity, specifically in the cardiometabolic cluster. To our knowledge, this is the first study examining the psychological impact of tea consumption across diverse disease contexts. Individuals without any chronic disease or with only one chronic disease did not exhibit this positive effect at the 3-year follow-up. Conversely, those with multimorbidity did. The most pronounced positive effect was observed in individuals with cardiometabolic multimorbidity, characterized by hypertension, heart disease, and DM (Cluster 5). The significance persisted even with the additional coexistence of CVD and respiratory disease. However, the association disappeared when cancer, dementia, Parkinson’s disease, peptic ulcer, and arthritis coexisted (Cluster 10). These findings are consistent with those of previous studies, which have demonstrated that tea consumption has a positive impact on individuals with cardiometabolic disease. Large Asian cohort studies have shown that tea consumption lowers the risk of cardiovascular (32) but not cancer (33, 34). This may explain why we observed that younger-old adults, who had an increasing prevalence of cardiometabolic multimorbidity as seen in prior evidence (35), significantly benefited from tea consumption compared with older-old counterparts. One possible explanation for this discrepancy is the higher burden of depressive and anxious symptoms among individuals with cancer (36), which is at least twice as high as that observed in individuals with cardiometabolic disease (37, 38). Such profound psychological distress experienced by cancer patients may be beyond the scope of the psychological benefit of tea to alleviate. Furthermore, since the majority of these studies were conducted within Asian populations, the generalization of these findings to western population would require further investigation. Notwithstanding these limitations, from a public health perspective, our findings underscore the preventive potential of habitual tea consumption, highlighting two critical implications (1): the value of establishing this health behavior earlier in life, and (2) the necessity for personalized approaches to enhance psychological resilience (PR) in aging populations, particularly given rising multimorbidity prevalence and individual variability in disease cluster presentations. Future longitudinal research should specifically investigate how both the age of initiation and cumulative duration of tea consumption modulate health trajectories across the lifespan.

The underlining mechanisms of the psychological benefits mentioned above may be attributed to sufficient L-theanine and polyphenols found in tea. L-theanine has been found to have relaxing and mood-enhancing properties (39, 40). Both animal and human studies have demonstrated its ability to suppress the hypothalamic-pituitary-adrenal-axis activity (41, 42), thereby promoting mental health. Additionally, epigallocatechin-3-gallate (EGCG), the most abundant polyphenols in tea, has been shown to mitigate excessive oxidative stress, which is linked to mitochondrial dysfunction, cellular senescence, and tissue inflammation, in the development of multimorbidity (43, 44). EGCG prevents these detrimental processes by directly scavenging reactive oxygen species and indirectly inhibiting the enzymes that promote oxidation through their effects on transcription factors (43, 45). Moreover, the biological effects of EGCG are shown to be concentration-dependent (46), which could explain why our main findings indicate that drinking tea daily yields more consistent results compared to other lower drinking frequencies. However, in pathological conditions characterized by excessive oxidative stress—particularly advanced cancer and neurodegenerative disorders—the therapeutic potential of dietary tea polyphenols appears limited by two key factors: first, the relatively low bioavailability of EGCG, and second, the inability to achieve sufficient tissue concentrations to counteract the dramatically elevated ROS production in these diseases (47, 48). These disease-stratified results suggest that tea’s health benefits are substantially modulated by underlying pathophysiology. Beyond these biological effects, the social benefits of tea consumption can also help improve PR as well. The duration of tea drinking provides more opportunities to chat, share, and connect with others. Such social engagement has been shown to improve metal health (49).

This study is the first to explore the longitudinal relationship between tea drinking habits and the dynamic change in PR in various disease contexts and provide insights for active health. However, it is important to note that several limitations exist. A key limitation lies in the incomplete assessment of tea consumption. Although the 2018 survey wave included documentation of tea types—such as green tea, red tea (black tea), oolong tea, white tea, yellow tea, dark tea (pu-erh tea), compressed tea, scented tea, and others—there was a lack of baseline data on tea type as well as information on consumption quantity and frequency. This limitation hindered our ability to conduct type-specific and dose-response analyses. This is particularly relevant given established variations in antioxidant composition across tea types (e.g., higher EGCG content in unfermented green tea versus catechins degradation in fermented black tea) (48, 50, 51). Therefore, future research should incorporate more detailed dietary records combined with biochemical validation of polyphenol exposure to better characterize underlying mechanisms. Second, since the survey was mostly self-reported, information bias is inevitable. Third, several subgroup analyses included only a few cases which might lead to low statistical power; therefore, our results should be interpreted with caution. Finally, future studies should consider the potential country differences in the relationship between tea consumption and PR.

## Conclusion

PR is crucial for older adults to cope with stress from chronic disease. Regular tea consumption may offer a cost-effective means to improve PR over time among older adults in the community, particularly those with cardiometabolic multimorbidity. This lifestyle may serve as a viable approach to promoting active health during late life stages. Additional research is required to explore the relationship between the type and dosage of tea consumed and their impact on PR.

## Data Availability

The original contributions presented in the study are included in the article/**Supplementary Material**. Further inquiries can be directed to the corresponding authors.
