# Supplementary material for: Tea consumption may improve psychological resilience among older adults with chronic diseases: a prospective cohort study
Source: Front Psychiatry. 2025 Jun 6;16:1594067. doi: 10.3389/fpsyt.2025.1594067 (PMC12179070; doi:10.3389/fpsyt.2025.1594067)
Supplement: Supplementary file 1 [file Table1.docx]

| Table S1: Measures of the psychological resilience scale. | | |
| --- | --- | --- |
| Number | Question of item | Scores based on responses |
| 1 | Do you feel the older you get, the more useless you are? | always=1;  often=2;  sometimes=3;  seldom=4;  never=5 |
| 2 | Do you often feel fearful or anxious? |  |
| 3 | Do you often feel lonely and isolated? |  |
| 4 | Do you always look on the bright side of things? | always=5;  often=4;  sometimes=3;  seldom=2;  never=1 |
| 5 | Can you make your own decisions concerning your personal affairs? |  |
