# Supplementary material for: Tea consumption may improve psychological resilience among older adults with chronic diseases: a prospective cohort study
Source: Front Psychiatry. 2025 Jun 6;16:1594067. doi: 10.3389/fpsyt.2025.1594067 (PMC12179070; doi:10.3389/fpsyt.2025.1594067)
Supplement: Supplementary file 2 [file Table2.docx]

Table S2. Demographics between the follow-up and lost to follow-up groups at baseline.

|  | | Overall n=26,454 | Follow-up group n=12,088 | Lost to follow-up group n= 14,366 |
| --- | --- | --- | --- | --- |
| Age (mean ± SD) | | 85.6 ± 12.0 | 80.3 ± 11. | 90.2 ± 10.9 |
| Sex, female (n (%)) | | 14729 (55.7) | 6259 (51.8) | 8470 (59.0) |
| Education, illiteracy (n (%)) | | 15945 (60.5) | 6492 (53.9) | 9453 (66.0) |
| Marital status (n (%)) | |  |  |  |
| Living with spouse | | 13036 (49.3) | 6924 (57.3) | 6112 (42.6) |
| Others | | 13396 (50.7) | 5152 (42.7) | 8244 (57.4) |
| Residential area (n (%)) |  | |  |  |
| Urban area | | 11541 (43.6) | 5005 (41.4) | 6536 (45.5) |
| Rural area | | 14913 (56.4) | 7083 (58.6) | 7830 (54.5) |
| Living arrangement (n (%)) |  | |  |  |
| With household member | | 21702 (82.1) | 10024 (83.0) | 11678 (81.4) |
| Alone/In institution | | 4734 (17.9) | 2057 (17.0) | 2677 (18.6) |
| Occupation (n (%)) | |  |  |  |
| Agriculture | | 16320 (61.9) | 7570 (62.9) | 8750 (61.0) |
| Non-agriculture | | 10055 (38.1) | 4472 (37.1) | 5583 (39.0) |
| Pension, yes (n (%)) | | 5055 (19.1) | 2606 (21.6) | 2449 (17.0) |
| Financial condition (n (%)) |  | |  |  |
| Sufficient | | 21356 (80.8) | 9770 (80.9) | 11586 (80.7) |
| Insufficient | | 5074 (19.2) | 2303 (19.1) | 2771 (19.3) |
| Smoking, yes (%) | | 5104 (19.3) | 2685 (22.2) | 2419 (16.9) |
| Alcohol consumption, yes (%) | | 5285 (20.0) | 2738 (22.7) | 2547 (17.8) |
| Exercising, yes (%) | | 8057 (30.5) | 4185 (34.7) | 3872 (27.0) |
| BADL score (mean ± SD) | | 6.8 ± 1.9 | 6.3 ± 1.0 | 7.2 ± 2.4 |
| IALD score (mean ± SD) | | 13.3 ± 5.7 | 10.9 ± 4.4 | 15.3 ± 5.9 |
| MMSE score (mean ± SD) | | 24.8 ± 5.9 | 26.5 ± 4.4 | 23.2 ± 6.6 |
| Zero chronic disease (n (%)) | | 14068 (53.2) | 6424 (53.1) | 7644 (53.2) |
| Single chronic disease (n (%)) | | 6472 (24.5) | 3009 (24.9) | 3463 (24.1) |
| Multimorbidity (n (%)) | | 5914 (22.4) | 2655 (22.0) | 3259 (22.7) |
| PRS (mean ± SD) | | 18.7 ± 3.2 | 19.23 (3.06) | 18.29 (3.18) |

BADL: basic activities of daily living; IADL: instrumental activities of daily living; MMSE: mini-mental state examination; PRS: psychological resilience score.
