# Supplementary material for: Tea consumption may improve psychological resilience among older adults with chronic diseases: a prospective cohort study
Source: Front Psychiatry. 2025 Jun 6;16:1594067. doi: 10.3389/fpsyt.2025.1594067 (PMC12179070; doi:10.3389/fpsyt.2025.1594067)
Supplement: Supplementary file 3 [file Table3.docx]

**Table S3.** Coefficients of tea consumption and PRS changes among the total sample.

|  | Inconsistent drinking β (95%CI) | | Consistent drinking β (95%CI) | Daily drinking β (95%CI) |
| --- | --- | --- | --- | --- |
| Female | | 0.023 (-0.206, 0.252) | 0.404 (0.088, 0.72)* | 0.518 (0.153, 0.884)* |
| Male | | 0.05 (-0.206, 0.306) | 0.121 (-0.173, 0.416) | 0.054 (-0.23, 0.338) |
| Younger-old (< 85 years) | | 0.234 (0.022, 0.446) * | 0.436 (0.177, 0.695) * | 0.337 (0.077, 0.596) * |
| Older-old (≥ 85 year) | | -0.231 (-0.516, 0.053) | -0.026 (-0.404, 0.351) | 0.121 (-0.296, 0.538) |
| No chronic disease | | -0.047 (-0.276, 0.183) | 0.248 (-0.042, 0.539) | 0.056 (-0.247, 0.359) |
| Single chronic disease | | 0.102 (-0.237, 0.44) | 0.138 (-0.3, 0.576) | 0.204 (-0.237, 0.645) |
| Hypertension | | 0.024 (-0.569, 0.617) | 0.28 (-0.441, 1) | 0.002 (-0.75, 0.755) |
| DM | | 1.259 (-1.041, 3.559) | -0.243 (-3.584, 3.099) | 0.945 (-2.396, 4.286) |
| Heart disease | | 0.118 (-1.001, 1.237) | 0.468 (-1.225, 2.161) | -0.312 (-1.675, 1.051) |
| CVD | | 0.402 (-1.377, 2.18) | -1.195 (-3.726, 1.336) | 0.26 (-2.043, 2.563) |
| Respiratory disease | | -0.171 (-0.971, 0.63) | 0.346 (-0.731, 1.423) | 0.522 (-0.532, 1.575) |
| Cancer | | 1.362 (-11.293, 14.017) | 6.116 (-12.072, 24.303) | 2.137 (-14, 18.274) |
| Peptic Ulcer | | 0.128 (-1.142, 1.397) | 0.742 (-0.904, 2.387) | -0.035 (-1.795, 1.725) |
| Parkinson's disease | | NA | NA | NA |
| Arthritis | | 0.063 (-0.648, 0.775) | -0.51 (-1.439, 0.419) | 0.155 (-0.818, 1.128) |
| Dementia | | NA | NA | NA |
| Multimorbidity | | 0.25 (-0.13, 0.63) | 0.456 (-0.001, 0.913) | 0.651 (0.182, 1.12) * |
| Cluster 1 | | 0.12 (-1.021, 1.262) | 0.359 (-1.004, 1.722) | -0.073 (-1.419, 1.274) |
| Cluster 2 | | 0.691 (-0.923, 2.305) | 0.653 (-1.67, 2.976) | 1.63 (-0.467, 3.726) |
| Cluster 3 | | 1.339 (-0.096, 2.774) | -0.285 (-2.417, 1.847) | 1.158 (-0.764, 3.08) |
| Cluster 4 | | 0.457 (-0.767, 1.682) | 0.939 (-0.578, 2.456) | 0.331 (-1.314, 1.977) |
| Cluster 5 | | 1.216 (-0.175, 2.606) | 2.035 (0.429, 3.641) * | 2.195 (0.295, 4.094) * |
| Cluster 6 | | 0.325 (-1.387, 2.036) | 1.412 (-0.79, 3.614) | 0.372 (-1.667, 2.412) |
| Cluster 7 | | -0.561 (-1.859, 0.736) | -0.777 (-2.677, 1.124) | -0.239 (-1.966, 1.487) |
| Cluster 8 | | -0.688 (-1.935, 0.559) | 0.271 (-1.146, 1.687) | 0.083 (-1.63, 1.796) |
| Cluster 9 | | 0.514 (-0.871, 1.899) | 1.568 (-0.019, 3.154) | 1.664 (0.088, 3.24) * |
| Cluster 10 | | 0.326 (-0.463, 1.115) | 0.436 (-0.45, 1.323) | 0.625 (-0.324, 1.573) |
| Cluster 1: hypertension and respiratory disease; Cluster 2: hypertension and peptic ulcer; Cluster 3: hypertension and CVD; Cluster 4: hypertension and heart disease; Cluster 5: hypertension, heart disease, and DM; Cluster 6: hypertension, heart disease, and arthritis; Cluster 7: hypertension, respiratory disease, and arthritis; Cluster 8: hypertension, heart disease, respiratory disease, arthritis, and peptic ulcer; Cluster 9: hypertension, heart disease, DM, CVD, and respiratory disease; Cluster 10: hypertension, DM, heart disease, CVD, respiratory disease, cancer, peptic ulcer, Parkinson’s disease, arthritis, and dementia; PRS: psychological resilience score; DM: diabetes mellitus; CVD: cerebrovascular disease. NA: not available. * *p* < 0.05. | | | | |
