# Supplementary material for: Tea consumption may improve psychological resilience among older adults with chronic diseases: a prospective cohort study
Source: Front Psychiatry. 2025 Jun 6;16:1594067. doi: 10.3389/fpsyt.2025.1594067 (PMC12179070; doi:10.3389/fpsyt.2025.1594067)
Supplement: Supplementary file 4 [file Table4.docx]

**Table S4.** Odd ratios of tea consumption and PR at 3-year follow-up among participants without severe cognitive impairment.

|  | Inconsistent drinking OR (95%CI) | | Consistent drinking OR (95%CI) | Daily drinking  OR (95%CI) |
| --- | --- | --- | --- | --- |
| Female | | 1.052 (0.918, 1.206) | 1.177 (0.978, 1.417) * | 1.272 (1.035, 1.564) * |
| Male | | 1.101 (0.943, 1.286) | 1.163 (0.973, 1.391) | 1.089 (0.917, 1.294) |
| Younger-old (< 85 years) | | 1.147 (1.012, 1.3) * | 1.288 (1.105, 1.501) * | 1.222 (1.05, 1.422) * |
| Older-old (≥ 85 year) | | 0.953 (0.799, 1.138) | 0.947 (0.752, 1.193) | 0.99 (0.773, 1.269) |
| No chronic disease | | 0.988 (0.862, 1.133) | 1.19 (1.004, 1.409) | 1.063 (0.892, 1.267) |
| Single chronic disease | | 1.164 (0.955, 1.417) | 1.089 (0.847, 1.4) | 1.159 (0.903, 1.488) |
| Hypertension | | 1.204 (0.85, 1.705) | 1.348 (0.89, 2.041) | 1.461 (0.951, 2.244) |
| DM | | 1.553 (0.345, 6.996) | 1.011 (0.108, 9.434) | 1.086 (0.133, 8.879) |
| Heart disease | | 0.794 (0.402, 1.568) | 0.728 (0.255, 2.074) | 0.809 (0.357, 1.832) |
| CVD | | 1.181 (0.419, 3.333) | 0.479 (0.11, 2.092) | 0.376 (0.089, 1.581) |
| Respiratory disease | | 1.379 (0.846, 2.247) | 1.277 (0.666, 2.449) | 1.481 (0.793, 2.767) |
| Cancer | | NA | NA | NA |
| Peptic Ulcer | | 1.183 (0.56, 2.497) | 2.116 (0.848, 5.28) | 1.196 (0.435, 3.292) |
| Parkinson's disease | | NA | NA | NA |
| Arthritis | | 1.073 (0.711, 1.62) | 0.68 (0.392, 1.179) | 0.853 (0.492, 1.479) |
| Dementia | | / | / | / |
| Multimorbidity | | 1.272 (0.991, 1.634) | 1.228 (0.904, 1.669) | 1.396 (1.033, 1.886) * |
| Cluster 1 | | 1.181 (0.613, 2.278) | 0.909 (0.418, 1.978) | 0.745 (0.344, 1.615) |
| Cluster 2 | | 0.915 (0.341, 2.454) | 0.711 (0.154, 3.271) | 1.46 (0.385, 5.536) |
| Cluster 3 | | 3.822 (1.556, 9.385) * | 1.277 (0.335, 4.868) | 2.145 (0.662, 6.95) |
| Cluster 4 | | 1.238 (0.577, 2.66) | 1.417 (0.557, 3.604) | 1.362 (0.515, 3.606) |
| Cluster 5 | | 2.582 (0.94, 7.094) | 5.481 (1.764, 17.032) * | 4.27 (1.131, 16.117) * |
| Cluster 6 | | 1.058 (0.412, 2.721) | 0.807 (0.234, 2.785) | 0.649 (0.21, 2.013) |
| Cluster 7 | | 1.033 (0.453, 2.356) | 1.942 (0.546, 6.901) | 1.454 (0.512, 4.13) |
| Cluster 8 | | 0.709 (0.313, 1.607) | 0.992 (0.405, 2.431) | 1.5 (0.527, 4.272) |
| Cluster 9 | | 1.323 (0.572, 3.059) | 2.075 (0.832, 5.178) | 2.472 (0.995, 6.145) |
| Cluster 10 | | / | / | / |
| OR: odd ratio; Cluster 1: hypertension and respiratory disease; Cluster 2: hypertension and peptic ulcer; Cluster 3: hypertension and CVD; Cluster 4: hypertension and heart disease; Cluster 5: hypertension, heart disease, and DM; Cluster 6: hypertension, heart disease, and arthritis; Cluster 7: hypertension, respiratory disease, and arthritis; Cluster 8: hypertension, heart disease, respiratory disease, arthritis, and peptic ulcer; Cluster 9: hypertension, heart disease, DM, CVD, and respiratory disease; Cluster 10: hypertension, DM, heart disease, CVD, respiratory disease, cancer, peptic ulcer, Parkinson’s disease, arthritis, and dementia; DM: diabetes mellitus; CVD: cerebrovascular disease. NA: not available. * *p* < 0.05. | | | | |
