# Supplementary material for: Tea consumption may improve psychological resilience among older adults with chronic diseases: a prospective cohort study
Source: Front Psychiatry. 2025 Jun 6;16:1594067. doi: 10.3389/fpsyt.2025.1594067 (PMC12179070; doi:10.3389/fpsyt.2025.1594067)
Supplement: Supplementary file 5 [file DataSheet1.pdf]

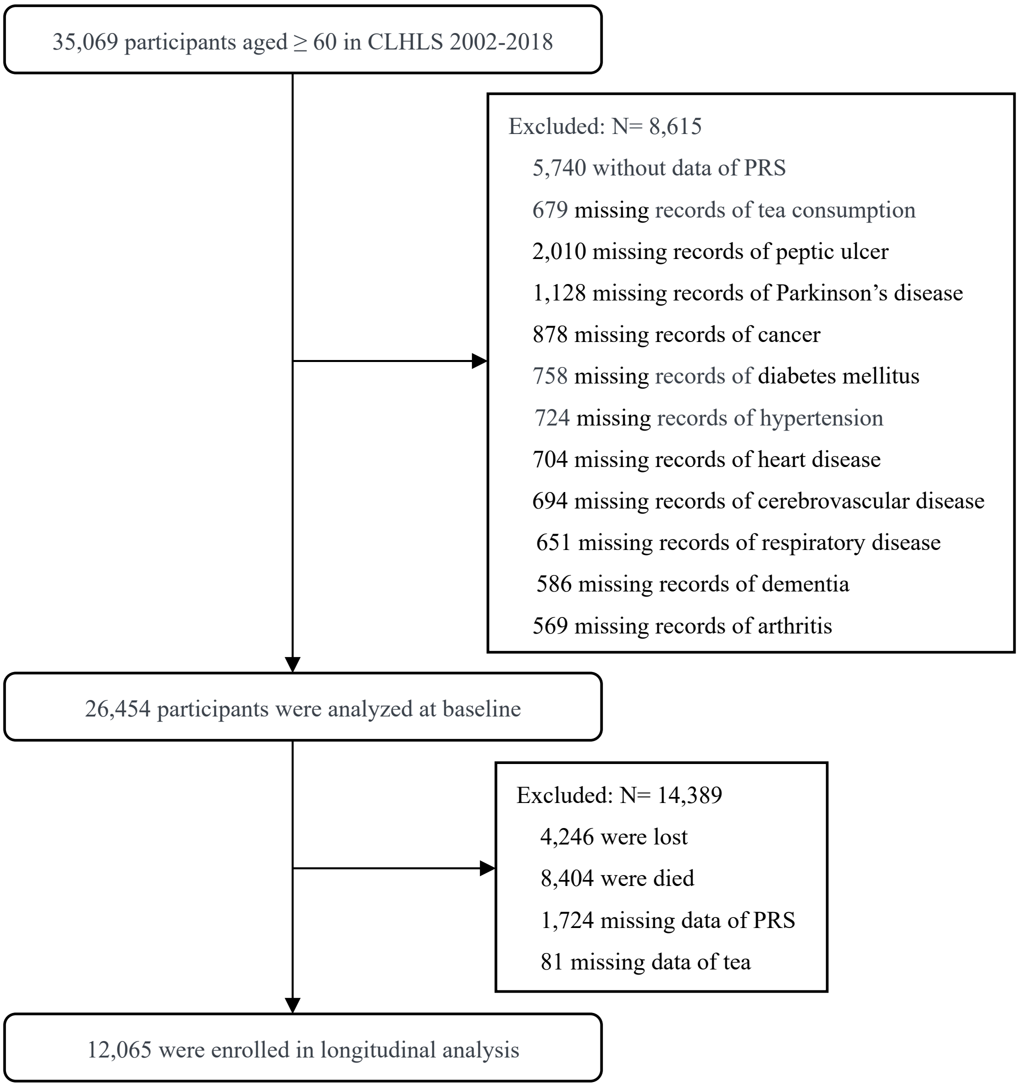

Figure S1. Flowchart of participants enrollment.

CLHLS: the Chinese Longitudinal Healthy Longevity Survey. PRS: psychological resilience score.
